# Iodine Supplemented Diet Positively Affect Immune Response and Dairy Product Quality in Fresian Cow

**DOI:** 10.3390/ani9110866

**Published:** 2019-10-25

**Authors:** Marco Iannaccone, Andrea Ianni, Ramy Elgendy, Camillo Martino, Mery Giantin, Lorenzo Cerretani, Mauro Dacasto, Giuseppe Martino

**Affiliations:** 1 Faculty of Bioscience and Technology for Food, Agriculture, and Environment, University of Teramo, Via R. Balzarini 1, 64100 Teramo, Italy; m.iannaccone@unina.it; 2 Department of Medical, Oral and Biotechnological Sciences, “G. d’Annunzio” University Chieti-Pescara, Via dei Vestini 31, 66100 Chieti, Italy; andreaianni@hotmail.it; 3 Department of Immunology, Genetics and Pathology, Uppsala University, Uppsala 75185, Sweden; ramy.elgendy@igp.uu.se; 4 Istituto Zooprofilattico Sperimentale dell’Abruzzo e del Molise “G. Caporale” Via Campo Boario, 64100 Teramo, Italy; c.martino@izs.it; 5 Department of Comparative Biomedicine and Food Science, University of Padua, Viale dell’Università 16, 35020 Legnaro (PD), Italy; mery.giantin@unipd.it (M.G.); mauro.dacasto@unipd.it (M.D.); 6 Pizzoli SPA, Via Zenzalino Nord, 40054 1 Budrio (BO), Italy; l.cerretani@pizzoli.it

**Keywords:** Iodine, immune response, oxidative stress, somatic cell count, fresh cheese, transcriptomics

## Abstract

**Simple Summary:**

Iodine represents an important micronutrient and plays a fundamental role in animal biology. This trace element is currently supplied to animal diet to investigate its potential effects on productive and reproductive performances. However, little is known about its role in the regulation of gene expression in ruminants. In this study, the dietary iodine supplementation in dairy cows showed effective modification of the expression of several molecular targets, with an improvement of the pathways involved in immune response and oxidative stress and undoubted positive repercussions on animal health.

**Abstract:**

The effects of iodine supplementation on the whole-transcriptome of dairy cow using RNA sequencing has been investigated in this study. Iodine did not influence the milk composition, while an improvement was observed in the immune response as well as in the quality of dairy product. Indeed, the iodine intake specifically influenced the expression of 525 genes and the pathway analysis demonstrated that the most affected among them were related to immune response and oxidative stress. As a consequence, we indirectly showed a better response to bacterial infection because of the reduction of somatic cell counts; furthermore, an improvement of dairy product quality was observed since lipid oxidation reduced in fresh cheese. Such findings, together with the higher milk iodine content, clearly demonstrated that iodine supplementation in dairy cow could represent a beneficial practice to preserve animal health and to improve the nutraceutical properties of milk and its derived products.

## 1. Introduction

Micronutrients are essential to orchestrate all physiological functions. Among them, iodine (I) plays a unique role because it is the main component of the thyroid hormones, i.e., thyroxine (T4) and triiodothyronine (T3) [1]. Since the thyroid gland regulates many metabolic processes, the extent of I requirement strongly depends on the age and stage of development of the individual [2]. Thus, when the I demand is not satisfied, reduced functionality of the thyroid gland could occur (hypothyroidism) with negative consequences for proper mental development, body growth, and fertility. For these reason, diet is often integrated with I supplements, generally provided through iodized salt [3]. For infants, milk constitutes the only I source; therefore, the dietary calibration of this micronutrients in dairy animals assumes relevant importance in order to obtain I-rich milk without inducing variations in animal performances [4].

In animal husbandry, I supplementation as calcium iodate, sodium iodide, and other iodine compounds is needed since the native iodine content of plant straight feed-stuffs is low; moreover, the increasing use of rapeseed meal (RSM) in livestock diets is associated with the intake of glucosinolates, which are known to be iodine antagonists inhibiting the activity of sodium iodide symporter [5]. For this reason, the European community has recently brought to 5 mg/kg the maximum level of I supplementation for milk-producing ruminants (milk intended for human consumption), while it remained at 10 mg/kg of complete feed for other ruminant categories [6]. Consequently, several studies have been carried out to evaluate the productive performances in animals fed the I supplementation. Weiss et al. [7] showed that I concentration increased in serum but not in milk after supplementation of this element in diets of dairy cows. In contrast, studies performed in small ruminants showed that I supplementation doubled the milk iodine content when compared with the control group, even though no evident effect was observed in the milk gross composition [8]. I supplementation showed also beneficial effects on healthy status. Indeed, early study on feedlot cattle demonstrated an increased resistance to foot rot [9], which is due to improved phagocytic cell function [10]. Moreover, it was shown in lambs that high-dose potassium iodide supplementation can be effective to decrease the severity of airway viral infections, supposedly through the augmentation of mucosal oxidative defenses [11,12].

Recently, the RNA-sequencing approach was shown to be useful to elucidate which molecular pathways are affected by I supplementation in sheeps [13] and showed the positive effects of olive pomace-supplemented diet on inflammation and cholesterol in laying hens [14]. However, to date, little is known about the effects of I supplementation on transcriptomic profiles in dairy cows. Moreover, we tried to correlate the information concerning the signaling pathways influenced by I supplementation, with the qualitative parameters of milk and derivatives, taking into account those studies in which it was shown that dietary iodine supplementation in ruminants contributes to an improvement in the quality of dairy products [15].

## 2. Materials and Methods

### 2.1. Animal and Study Design

The study design was approved by the Teramo University Institutional Animal Care and Use Committee. Animals were managed according to Directive 2010/63/EU of the European Parliament regarding the protection of animals used for experimentation or other scientific purposes [16].

Twenty-two Friesian cattle, homogenous for age (range between 39 and 42 months), number of births (2 calves), and lactation length (70 ± 5 days), have been enrolled in this study. Animals, belonging to the same farm and bred in the same way were randomly divided in 2 groups of 11 cows each. During the 3-week acclimatization period (21 days), both the control (CTR) and the experimental iodine groups (IG) received a basal diet that mainly consisted of alfalfa hay plus a custom-formulated concentrate supplemented with 20 mg/day/animal I in order to guarantee the daily micronutrient requirement for each animal; then, the IG animals were fed for 56 days (during April and May) with a custom-formulated concentrate supplemented with additional 65 mg/day/animal of I in order to obtain a total intake of about 85 mg; this amount has been set not to exceed the maximum level allowed by law [17]. Ingredients and composition of total mixed ration (TMR) administered to the animals during the experimental period are reported in Table 1.

### 2.2. Blood and Milk Sampling

Individual whole blood (WB) samples were collected at the beginning (T0) and at the end of the dietary supplementation for the evaluation of the hematochemical parameters. In the case of the RNA-Seq analysis, 2.5 mL of jugular venous blood was collected in duplicate from each animal only at the end of the experimental period. In this case, samples were collected in PAXgene™ tubes (Qiagen SpA, Milan, MI, Italy), stored overnight at room temperature, and then placed at −20 °C until RNA isolation, following the manufacturer’s instructions.

Regarding milk, at the beginning (T0) and after 8 weeks (T8) of dietary supplementation, 50 mL of individual samples was collected from each group in triplicate in 80 mL polypropylene tubes containing a bronopol/sodium azide preservative solution, useful to extend the storage time before the analysis up to 48–72 h. Milking was performed through mechanical support (DeLaval, Milan, Italy), and teat disinfection occurred both before and after milking by using iodine-free solutions (DeLaval, Milan, Italy).

### 2.3. Blood Analysis

Complete blood cell count with leukocyte formula (total white blood cells, monocyte, lymphocyte, basophils, neutrophils, and eosinophils) for both the CTR and IG groups at T0 and T8 were performed at the Veterinary and Public Health Institute (Teramo, Italy) using a laser-based hematology analyzer with software applications for animal species (ADVIA 120 hematology system, Siemens, Munich, Germany). Plasma samples were analyzed for thyroid hormones (Thyroid-stimulating hormone (TSH), T3, and T4) with an automatic biochemistry analyzer (ILAB 650, Instrumentation Laboratory-Werfen, Milan, Italy) and following the routine procedure of the institute (Veterinary and Public Health Institute “G. Caporale”, Teramo, Italy).

### 2.4. Chemical Analysis of Milk

Chemical composition of milk (fat, protein, casein, lactose, and urea) was determined by MilkoScan FT 6000 (Foss Integrator IMT; Foss, Hillerød, Denmark), whereas the somatic cells count (SCC) was performed using the Fossomatic FC (Foss).

### 2.5. Iodine Determination in Milk

The amount of I in the milk at T0 and T8 was determined according to Fecher et al. (1998) with some modifications [18]. Briefly, for each sample, 0.5 g of milk were homogenized with tetramethylammonium hydroxide (0.25 M) and 2 mL of deionized water (30%). Then, samples were heated in a microwave (800 W) at 170 °C for 30 min. After cooling, samples were transferred into a sterile tube and diluted with distilled water to a final volume of 15 mL. After centrifugation at 12,000 rpm × min^−1^ at room temperature for 10 min, samples were filtered through polytetra-fluoroethylene (PTFE) syringe filters (0.45 μm) and, finally, stored at 4 °C until analysis. A standard calibration curve was created using six calibration points equals to concentrations of 0, 5, 10, 25, 50, and 100 mg/L of I in tetramethylammonium hydroxide. For the carrier and gas formation, argon gas was used at flow rates of 1.05 and 0.2 L/min, respectively. The iodine content was determined by an inductively coupled plasma mass spectrometer Agilent 7500ce ICP-MS (Agilent Technologies, Palo Alto, CA, USA) at m/z = 127 and a total acquisition time of 21 s. Before the sequence analysis, the ICP-MS was auto-tuned by a solution containing 1 ppb of different metals (Li, Y, Ce, Tl, and Co).

### 2.6. Library Preparation and RNA-Seq Analysis

Total RNA was isolated from blood using the PAXgene blood RNA kit (Qiagen, Milan, Italy) as per the manufacturer’s instructions. Total RNA concentration was determined by the NanoDrop ND-1000 spectrophotometer (NanoDrop Technologies Inc., Wilmington, DE, USA), and its quality was measured by the 2100 Bioanalyzer and RNA 6000 Nano kit (Agilent Technologies, Santa Clara, CA, USA). Strand-specific RNA-Seq libraries were prepared using the SureSelect strand-specific mRNA library preparation kit (Agilent Technologies, Santa Clara, CA, USA) as per the manufacturer’s protocol. In brief, poly(A) RNA was purified from 1µg of total RNA using two serial rounds of binding to oligo(dT) magnetic particles and, then, fragmented and reverse transcribed to generate cDNA. Illumina-specific adaptor was sequentially ligated to the 3’ end of cDNA fragments and purified using the AMPure XP beads (Beckman Coulter, Brea, CA, USA), and finally PCR-amplified (13 cycles) using an appropriate indexing primer to allow further samples multiplexing. The PCR-amplified libraries were purified with the AMPure XP beads (Beckman Coulter, Brea, CA, USA) and then assessed for their quality and fragments distribution using the 2100 Bioanalyzer DNA 1000 assay (Agilent Technologies, Santa Clara, CA, USA). In the presence of adaptor-dimers (Electropherogram’s peak at 100 to 150-bp), another round of magnetic beads purification was performed. Libraries were quantified by both the Qubit^®^ Fluorometer (Life Technologies, Carlsbad, CA, USA) and the qPCR-based NEBNext library quantification kit (New England BioLabs, Hitchin, UK). Finally, libraries were pooled and then sequenced by an Illumina HiSeq 2500 for 50 sequencing cycles.

The raw 50-bp single-end sequences (Sanger/Illumina 1.9 encoding) were quality controlled using FastQC (v.0.11.4; Babraham Institute, Cambridge, UK), and the low-quality bases (quality scores < 30) and adaptor contamination (if present) were removed by Trimmomatic v.0.36. The high-quality reads were mapped by HISAT v.2.0.5 against the Bos taurus reference genome (Ensembl Bos_taurus_UMD_3.1.1). The uniquely mapped reads aligned to exons were counted with HTSeq v.0.6.1 [19] and then tested by the DESeq2 R package v.1.14.1 [20] for the presence of differentially expressed genes (DEGs) in the IG group compared with the CTR one (e.g., T8 I vs T8 CTR). Genes with a false discovery rate (FDR) less than 0.05 were considered as DEGs. All analyses were performed using the software Artificial Intelligence RNA-seq (A.I.R; developed by Sequentia Biotech, Barcelona, Spain) and the sequencing data (FASTQ files) associated with this project are deposited in the GenBank’s Sequence Read Archive (SRA) under the accession number PRJNA516565.

### 2.7. Enriched Pathway Analysis

The STRING software (Version 11.0, http://string-db.org/) was used to identify canonical pathways using the dataset of 525 DEGs identified between the IG group and the CTR one with a FDR < 0.05. We set the interaction score as 0.9, the highest value permitted by the software to avoid false positives. The significance of the canonical pathway was measured with the *p*-value and the ratio of DEG/number of genes in the pathway.

### 2.8. Ricotta Cheese-Making Procedure and Lipid Oxidation by Thiobarbituric Acid Reactive Substance Test

At the end of the supplementation period, an aliquot of about 150 L of bulk milk from each experimental group was separately collected and manipulated according to common cheese-making procedures. The whey separately collected from each experimental group during the cheese-making was used to produce “ricotta” cheese. This was obtained by acid-thermal coagulation of whey, through heating up to 80 °C and adding 50 g of lactic acid. The whey flocs were collected in holed baskets with a total capacity equal to 0.5 L and left to drain for 30 min. Then, baskets still containing the ricotta were transferred in a refrigerator and stored under chilling conditions (4 °C). From each experimental group, 9 ricotta forms (single weight = about 400 g) were obtained. In order to evaluate lipid peroxidation, thiobarbituric acid reactive substances (TBARS) were measured at day 0 and day 7. The analysis was performed according to the procedure reported by Ianni et al. with slight modifications [21]: 4.5 g of frozen ricotta cheese were mixed within 2 min of sample withdrawal from the freezer, with 450 μL of 0.1% of butylated hydroxytoluene in methanol with the aim to stop the oxidation process. The mixture was homogenized with UltraTurrax T-25 high speed homogenizer (IKA, Staufen, Germany) in 40 mL of an aqueous solution of 7% trichloroacetic acid and, then, distilled; 1.5 mL of each distillate was mixed with an equal volume of a 0.02 M thiobarbituric acid (TBA) in 90% acetic acid, and the solution was kept for one hour in a thermostatic bath at 80 °C. Only after cooling, the absorbance at 534 nm was evaluated with a spectrophotometer (JENWAY 6305 UV/vis, Jenway, Essex, UK). The amount of malondialdehyde (MDA) of each sample was calculated by using a calibration curve ranging from 0 to 100 ppm (R^2^ = 0.986), and results were expressed in µg of MDA per g of cheese.

### 2.9. Statistics

GraphPad Prism version 6.0 (GraphPad Software, La Jolla, CA, USA) was used for statistical analysis. Differences in milk parameters, as well as the amount of thyroid hormone in blood sera and malondialdehyde (MDA) concentrations in ricotta fresh cheese were statistically evaluated by using ordinary two-way ANOVA. 

## 3. Results

### 3.1. Serum Thyroid Hormone and Iodine Concentrations

All animals maintained a good state of health for the entire duration of the trial, and no significant variations in milk yield have been observed between the two groups at the end of the experimental period. 

**Figure 1:**
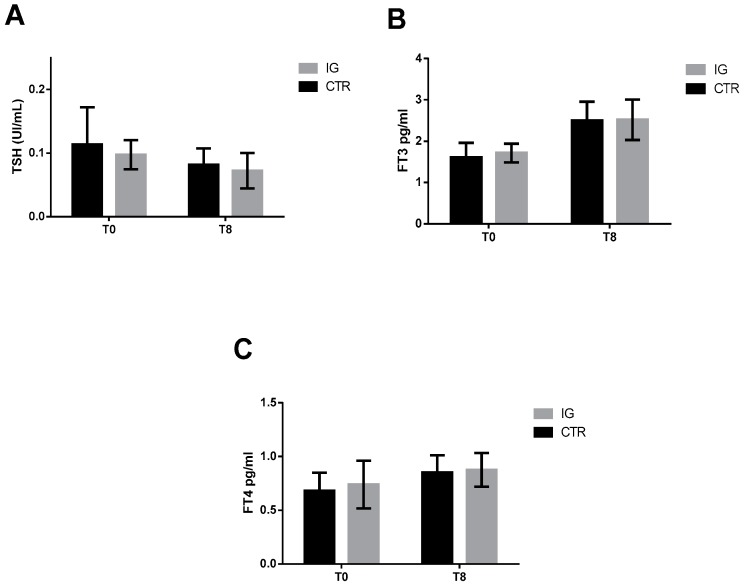
Thyroid hormone concentrations: Thyroid-stimulating hormone (**A**), free thyroxine (**B**), and free triiodothyronine (**C**) were measured in serum samples from control (CTR) (*n* = 11) and iodine group (IG) (*n* = 11) at the beginning and at the end of iodine supplementation. Data are expressed as mean ± SD, and differences were assessed using 2-way ANOVA.

Because I affects production and secretion of thyroid hormones, we quantified the levels of thyrotropin (TSH), triiodothyronine (T3), and thyroxine (T4) at the beginning (T0) and at the end (T8) of the supplementation in the serum blood samples. As shown in Figure 1, no differences in hormone levels were ever noticed between groups, suggesting that the thyroid functionality was not affected by the iodine supplementation and that the CTR group was not in iodine-deficiency condition.

**Figure 2:**
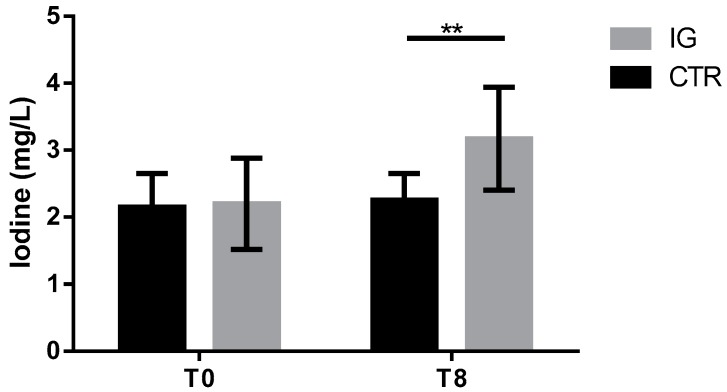
Iodine quantification in milk samples: Iodine was quantified in milk samples of both CTR (*n* = 11) and IG (*n* = 11) at the beginning and at the end of iodine supplementation. Data are shown as mean ± SD, and differences were assessed using 2-way ANOVA. ** *p*-value < 0.01.

As expected (Figure 2), we appreciate a higher amount of iodine in the I group, indicating that consumption of milk from dairy cows fed with high iodine intake is helpful to integrate diets where physiological stages like infancy and/or pregnancy require higher iodine intake [22].

However, it is worth mentioning that milk parameters were not influenced by iodine supplementation (Table S1).

### 3.2. Influence of I-supplemented Diet on Blood Transcriptome

To identify the molecular networks associated to I supplementation, we collected peripheral blood from both groups at the end of I supplementation period and performed a transcriptomic analysis by RNA-Seq. After trimming and quality control, on average, 96% of reads resulted of high-quality reads (Table S2) and were mapped against Bos taurus reference genome (Bos_taurus_UMD_3.1.1). 

**Figure 3:**
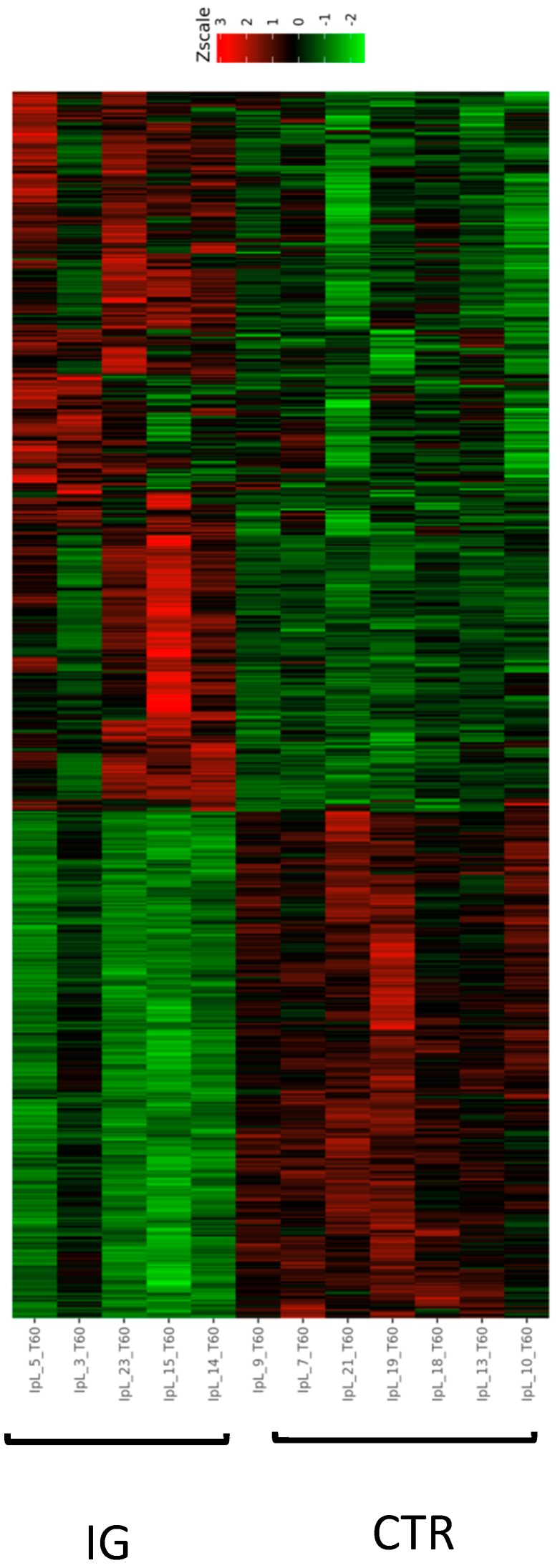
Heat map analysis of the 525 differentially expressed genes identified at the end of iodine supplementation period.

**Figure 4:**
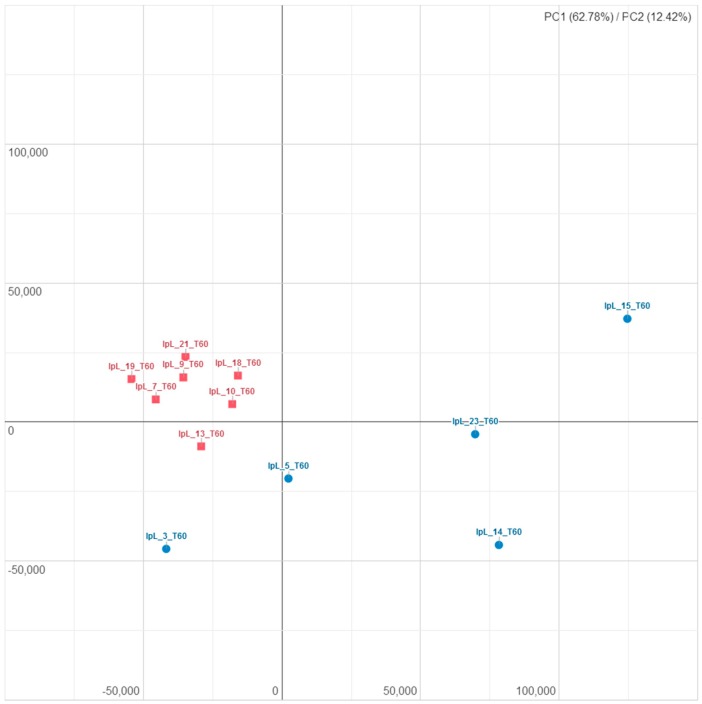
Principal components analysis using the 525 differentially expressed gene identified at the end of the iodine supplementation period: The blue circles identify the iodine samples while the red squares identify CTR samples.

Filtering our data by using a FDR < 0.05, we identified 525 DEGs (Table S3); in particular, 274 and 248 genes were respectively down- and upregulated in the I group compared to CTR; moreover, they enabled us to discriminate the two groups not only on a heat map scale but also on a hierarchical clustering analysis, thereby indicating the robustness of analysis (Figure 3 and Figure 4).

In order to identify the enriched pathways and putative interactions among DEGs, we interrogated the STRING software by using the up- and downregulated genes. To increase stringency and confidence in our data set, we applied the highest available interaction score (0.9), and in Table 2 (downregulated) and Table 3 (upregulated) are listed the most significant pathways (FDR < 0.05). The highest enriched pathway was “Fc gamma R-mediated phagocytosis” (FDR: 2.63 × 10^−6^), which was associated with clearance of opsonized particles, suggesting that iodine supplementation could help the immune system against invading bacteria [23]. Interestingly, also, the “oxidative phosphorylation” pathway was dysregulated by iodine supplementation, indicating a positive effect of iodine on mitochondrial activity with consequent reduction of oxidative stress, which could potentially act on quality dairy products.

### 3.3. Effects of I-Supplementation on Quality of Dairy Products

**Figure 5:**
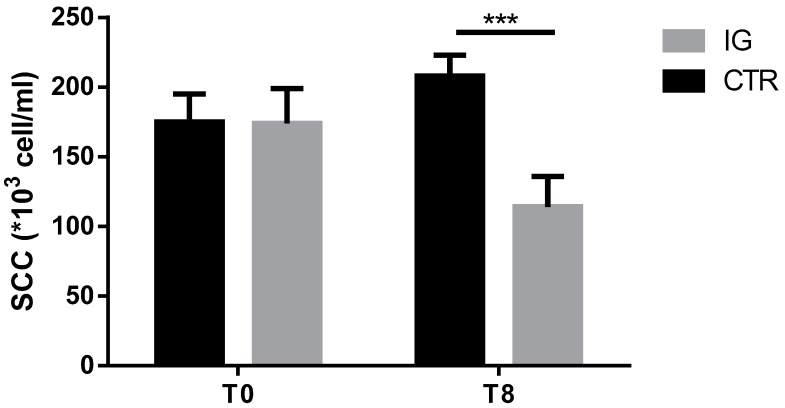
Somatic cell count in individual milk samples from CTR (*n* = 11) and IG (*n* = 11): Data are shown as mean ± SD, and differences were assessed using 2-way ANOVA. *** *p*-value < 0.001.

To test the hypothesis if I supplementation could positively influence quality of dairy products, we evaluated the milk somatic cell count (SCC) at the beginning T0 and the end T8 of the supplementation. Clearly, in Figure 5, we demonstrated a significant reduction of SCC, indicating that the I treatment ameliorated immune response against bacteria responsible for infectious diseases like mastitis [24].

**Figure 6:**
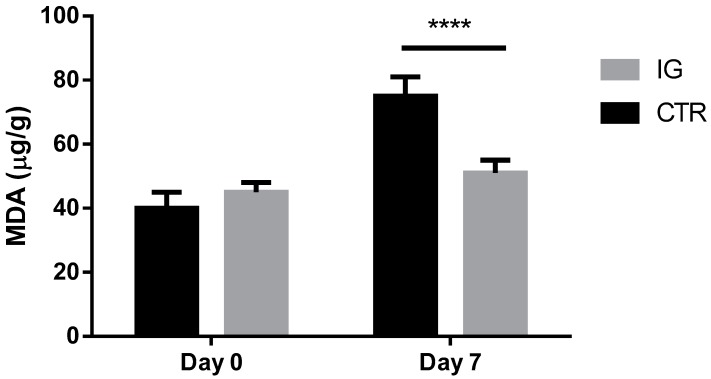
Malondialdehyde (MDA) levels at 0 (40 ± 5 vs. 45 ± 3) and 7 (75 ± 6 vs. 51 ± 4) days from fresh cheese ricotta stored at 4 °C: Data represent mean ± SD, **** *p* < 0.0001, two-way ANOVA (5 samples/group).

Then, we evaluated also the quality of fresh dairy cheese (ricotta) obtained using milk derived by the CTR and I groups. Lipid oxidation is strongly influenced by the redox homeostasis, and in our data set, iodine influences mitochondrial activity, which is highly involved in controlling oxidative stress. Thus, we measured the time-dependent variation (at day 0 and day 7) in malondialdehyde (MDA) levels in fresh ricotta cheese kept at 4 °C. As showed in Figure 6, ricotta from the I group showed statistically lower amounts of MDA, which confirms the favorable effect of I supplementation on cheese quality.

## 4. Discussion

In this study, we provide evidence that dairy cows fed an I-supplemented diet for a limited time showed transcriptional changes related to immune response and oxidative stress. In our experience, whole blood is a good starting point to understand in ruminants the effects of different diet supplements such as agro-industrial by-products and microelements [25,26,27]. To avoid that differences identified in gene expression in this study that could be influenced by different composition in white blood cell, we measured the complete blood cell count both in CTR and IG at the beginning T0 and at the end of supplementation, and we did not find any difference (Table S4). Then, because I is the major component of thyroid hormones, we measured the free hormone levels in the sera of both groups. In agreement with previous studies, the thyroid hormone levels did not differ between the two groups, clearly indicating that I supplement used in this study does not affect thyroid functionality [28].

In our study, RNA-sequencing analysis confirmed that I supplementation deregulated the expression of numerous genes, showing a significant biological connection confirmed by a very small *p*-value for protein–protein interaction, indicating no random nodes within our data set (PPI enrichment *p*-value < 1.0 × 10^−16^). More in detail, we identified many pathways related to immune response (Table 2), and the most significant one was that of “Fc gamma R-mediated phagocytosis” (FDR: 2.36 × 10^−6^) and, as a consequence, also of “B cell receptor signaling pathway” (FDR: 0.0024), which is in agreement with previous study in which I exposure produced an increase in immunoglobulin synthesis by lymphocytes [29]. Moreover, phagocytes are the lymphocyte subsets which express the higher level of sodium iodide symporter [28]. Thus, iodide supplementation reinforces immune response via strengthened antibody production and phagocytosis. Moreover, iodide also interacts with myeloperoxidase and H2O2, reinforcing the killing of bacterial activity [30]. Altogether, these positive effects are potentially associated with pathogen clearance and we have demonstrated that milk collected from animals belonging to the experimental group showed a reduced somatic cell count, which has directly associated with reduced bacterial load [24].

Also, oxidative phosphorylation is one of the activated pathways in our study (4.75 × 10^−4^). The effects of I and thyroid hormones have been previously fully reviewed [31]; thus, it is not surprising to find the activation of genes belonging to the multimeric protein ATP synthase (ATP5G2 and ATP5D), which is responsible for ATP production from ADP. Indeed, it is been demonstrated that increased production of ATP was inversely associated with production of cellular damage enzyme associated biomarkers (Serum lactate dehydrogenase, LHD; creatine phosphokinase, CPK; Alkaline phosphatase, ALP) following I supplementation. Also, several genes encoding for different subunits of the NADH: ubiquinone oxidoreductase were upregulated in our data set. These results further confirm the effect of I-supplementation on oxidative phosphorylation with a potential favourable energy balance.

In addition, I has been considered, in an evolutionary sense, one of the most ancient antioxidant because it can easily interact with reactive oxygen species (ROS) [32]. In our study, milk samples collected from the experimental groups have shown increased I content in agreement with previous studies on I supplementation in ruminants [13,33]. Thus, when we analyzed ricotta cheese produced by using milk from the CTR and I groups, we appreciated a reduced production of MDA in IG & CTR, indirectly confirming a reduction in lipid oxidation. 

In conclusion, in our study, we have demonstrated that iodine supplementation has multiple positive effects on animal health and dairy products quality. Indeed, on one side, I supplementation ameliorates immune response and resistance to infectious disease, improving phagocytosis, and on the other side, it improves the quality of fresh cheese, reducing lipid oxidation with beneficial effects also on consumer’s health. Moreover, because of the higher I content in milk, make potentially this product suitable also for subjects that, for particular physiological conditions (i.e., pregnancy) or life stage (i.e., infant), need more I intake.

## Figures and Tables

**Table 1:** Ingredients and composition of total mixed ration (TMR) administered to each animal of both group of study.

**Ingredients of TMR**	
Corn silage, %	23.7
First cut, alfalfa hay, %	5.3
Corn meal, %	3.4
Soybean, meal, %	3.2
Fine bran, %	3.0
Barley, meal, %	1.9
CaCO_3_, %	0.2
Vitamins and minerals, %	0.4
Kg of dry matter/head per day	22.41
**Chemical Composition of TMR**	
Dry Matter, %	56.76
Crude protein ^1^, %	15.34
Ether extract ^1^, %	2.97
Ash ^1^, %	5.31
Neutral detergent fiber ^1^, %	32.51
Acid detergent fiber ^1^, %	20.03
Starch ^1^, %	27.02
Iodine (mg/head/day) ^1^	20 (+65) *

^1^ On a dry matter (DM) basis; * In brackets is the amount of iodine added to the diet for the experimental group.

**Table 2:** List of significantly enriched pathways obtained using the downregulated genes following iodine supplementation.

Pathway	FDR	Genes
regulation of transcription, DNA-templated	1.30 × 10^−5^	ZNF93, SBNO1, ZBTB6, SNAPC3, ZFX, ZNF12, LOC530973, ZNF655, LOC104968476, ZNF175, MAP3K7, ZNF184, JADE1, ZNF182, ZNF148, RBAK, ZNF248, ZNF286A, DNTTIP2, MYNN, ZNF605, ZNF572, ZNF436
cellular response to DNA damage stimulus	7.45 × 10^−4^	SHPRH, HELB, ZMAT3, FMR1, RNF168, SPRTN, USP16, NEK4
transcription, DNA-templated	0.0057	CCNT2, ZKSCAN8, ZBTB6, SNAPC3, ZNF131, ZFX, SCAI, ZNF518A, MAP3K7, ZNF184, JADE1, ZNF148, PSIP1, DNTTIP2, USP16, MYNN, ZNF572
RNA processing	0.0153	DHX29, U2SURP, YTHDC2, DHX36
negative regulation of transforming growth factor beta receptor signaling pathway	0.0186	ZNF451, LEMD3, SMURF2, SIRT1
cell division	0.0473	CDC40, USP37, USP16, CENPJ, SPICE1, SMC3

FDR: false discovery rate.

**Table 3:** List of significantly enriched pathways obtained using the upregulated genes following iodine supplementation.

Pathway	FDR	Genes
Fc gamma R-mediated phagocytosis	2.63 × 10^−6^	AKT1, AKT2, ARF6, ARPC1B, CFL1, CRKL, GSN, LIMK1, RAC2, SCIN, VASP
Non-alcoholic fatty liver disease (NAFLD)	1.33 × 10^−5^	AKT1, AKT2, COX8A, GSK3A, MAP3K11, NDUFA7, NDUFB7, NDUFS6, NDUFS7, NDUFS8, SDHA, TGFB1, UQCR11
Oxidative phosphorylation	0.0005	ATP5D, ATP5G2, COX8A, NDUFA7, NDUFB7, NDUFS6, NDUFS7, NDUFS8, SDHA, UQCR11
Rap1 signaling pathway	0.0008	ACTB, ACTG1, AKT1, AKT2, CRKL, MAP2K3, RAC2, RAPGEF1, RASSF5, SIPA1, TLN1, VASP
Bacterial invasion of epithelial cells	0.0016	ACTB, ACTG1, ARPC1B, CRKL, PXN, RHOG, SEPT9
Proteasome	0.0078	PSMB10, PSMB4, PSMB8, PSMB9, PSMD4
Carbon metabolism	0.0079	ENO1, GPI, HK1, IDH2, PFKL, SDHA, TPI1
Focal adhesion	0.0079	ACTB, ACTG1, AKT1, AKT2, CRKL, PXN, RAC2, RAPGEF1, TLN1, VASP
Regulation of actin cytoskeleton	0.0079	ACTB, ACTG1, ARPC1B, CFL1, CRKL, GSN, LIMK1, PXN, RAC2, SCIN
B cell receptor signaling pathway	0.0024	AKT1, AKT2, CD81, NFKBIB, RAC2

FDR: false discovery rate.

## Supplementary Materials

The following are available online at https://www.mdpi.com/2076-2615/9/11/866/s1, Table S1: Milk parameter comparison between control and I-supplemented group, Table S2: Quality control of reads after trimming, Table S3: Complete list of all significant DEGs following the iodine supplementation period, Table S4: Blood cell count comparison between control and I supplementation group.
