# Supplementary material for: Iodine Supplemented Diet Positively Affect Immune Response and Dairy Product Quality in Fresian Cow
Source: Animals (Basel). 2019 Oct 25;9(11):866. doi: 10.3390/ani9110866 (PMC6912399; doi:10.3390/ani9110866)
Supplement: Supplementary file 1 [file animals-09-00866-s001.pdf]

Supplementary Table 1. Milk parameters comparison between control and I-supplemented groups

Parameters	CTR		IG	
	**T0**	**T8**	**T0**	**T8**
Fat (%)	3.61±0.36	3.47±0.60	3.79±0.71	3.66±0.69
Protein (%)	3.08±0.23	3.11±0.26	3.11±0.28	3.12±0.24
Lactose (%)	4.87±0.07	4.67±0.40	4.77±0.24	4.85±0.15
Caseins (%)	2.47±0.18	2.40±0.18	2.47±0.18	2.46±0.19
Urea (ppm)	19.47±2.99	20.88±4.51	16.99±5.79	19.86±3.37
pH	6.65±0.07	6.51±0.07	6.70±0.06	6.52±0.04

Supplementary Table 2. Quality control of reads after trimming.

Sample	Species	Reads before data quality control	Reads after data quality control	%
IpL_1_CTR	*Bos taurus*	18771111	18101909	96,43494
IpL_14_CTR	*Bos taurus*	15486569	14885274	96,11731
IpL_15_CTR	*Bos taurus*	19965675	19271481	96,52306
IpL_17_CTR	*Bos taurus*	15904541	15051300	94,63524
IpL_23_CTR	*Bos taurus*	15343479	14737496	96,05055
IpL_3_CTR	*Bos taurus*	28384989	26777582	94,33712
IpL_5_CTR	*Bos taurus*	25386691	24284609	95,65882
IpL_10_I	*Bos taurus*	19963230	19351452	96,93548
IpL_13_I	*Bos taurus*	19961303	19303620	96,70521
IpL_16_I	*Bos taurus*	26889603	25480848	94,76097
IpL_18_I	*Bos taurus*	29147467	27798053	95,37039
IpL_19_I	*Bos taurus*	23535762	22517344	95,67289
IpL_21_I	*Bos taurus*	19323387	18390965	95,17465
IpL_7_I	*Bos taurus*	17152603	16330978	95,20991
IpL_9_I	*Bos taurus*	23607485	22601995	95,7408

Supplementary table 3. Complete list of all significant DEGs following the iodine supplementation period

Gene name	logFC	FDR	Gene description
AASDH	0,41181084	0,003156107	
ACAD11	0,291201852	0,046363247	Acyl-Coenzyme A dehydrogenase family, member 11 [Uniprot Acc. Q0P5G8];
ACADSB	0,278549458	0,046458864	Short/branched chain specific acyl-CoA dehydrogenase, mitochondrial [Uniprot Acc. Q5EAD4];
AKAP11	0,22826178	0,013406958	
AKAP9	0,252613225	0,014122844	
ANGEL2	0,272334236	0,02487718	Protein angel homolog 2 [Uniprot Acc. A6H7I3];
ANKRD28	0,196560976	0,023206764	
AP5M1	0,354195382	0,001658985	AP-5 complex subunit mu-1 [Uniprot Acc. Q5E9X5];
APPL1	0,271632408	0,018097212	APPL1 protein [Uniprot Acc. A5PKI0];
ASTE1	0,333841314	0,02469778	
ATG14	0,245320115	0,036894712	Uncharacterized protein [Uniprot Acc. E1BAQ5];
ATL2	0,27942771	0,030302774	Uncharacterized protein [Uniprot Acc. E1BE28];
ATM	0,383118278	0,030458606	
ATR	0,297089126	0,004916491	
ATRX	0,288260724	0,023926585	
BDP1	0,372836921	0,006242876	
BLOC1S6	0,324401482	0,008391311	Biogenesis of lysosome-related organelles complex 1 subunit 6 [Uniprot Acc. Q08DU8];
BROX	0,220356711	0,048199854	
C2H2orf69	0,337052026	0,030279575	
C7H5orf24	0,284108549	0,030290693	
CAPRIN2	0,365545783	0,034608365	
CASP8AP2	0,297038304	0,018097212	Uncharacterized protein [Uniprot Acc. E1BLU4];
CCDC15	0,283510603	0,025564133	
CCDC66	0,485958277	0,001187	Uncharacterized protein [Uniprot Acc. F1MLR5];
CCNT2	0,274506108	0,015616764	Uncharacterized protein [Uniprot Acc. E1B6Z5];
CDC40	0,252766756	0,017890047	Cell division cycle 40 homolog (S. cerevisiae) [Uniprot Acc. Q0P5H3];
CENPC	0,323421618	0,006242876	Uncharacterized protein [Uniprot Acc. E1BAK5]; Centromere protein C 1 [Uniprot Acc. Q2KJ76];
CENPJ	0,337593365	0,026346998	Uncharacterized protein [Uniprot Acc. E1BL95];
CEP135	0,316529645	0,023135536	
CEP290	0,277731188	0,037485753	Centrosomal protein of 290 kDa [Uniprot Acc. Q9TU23];
CEP295	0,333884591	0,001187	

CEP85L	0,304744881	0,010802279	
CEP97	0,321784393	0,046342544	Uncharacterized protein [Uniprot Acc. E1BBI2];
CHD9	0,26377541	0,01917867	Uncharacterized protein [Uniprot Acc. E1BDZ3];
CHML	0,356787575	0,024376248	Uncharacterized protein [Uniprot Acc. F1N2I0];
CMTR2	0,332685187	0,009866319	Uncharacterized protein [Uniprot Acc. G3MZP6];
COG6	0,244644104	0,026168373	Conserved oligomeric Golgi complex subunit 6 [Uniprot Acc. Q3SZI7];
CTCF	0,159341613	0,038491543	CCCTC-binding factor (Zinc finger protein) [Uniprot Acc. Q08DH9];
CTDSPL2	0,222695665	0,008989393	Uncharacterized protein [Uniprot Acc. E1B8W1];
DHX29	0,242032932	0,012284834	
DHX36	0,220835785	0,022224422	DEAH (Asp-Glu-Ala-His) box polypeptide 36 [Uniprot Acc. Q05B79];
DNTTIP2	0,245458289	0,04650335	Deoxynucleotidyltransferase terminal-interacting protein 2 [Uniprot Acc. Q0P5H2];
EDRF1	0,192722418	0,023299403	
ELF2	0,208363028	0,007014962	Uncharacterized protein [Uniprot Acc. F1N599];
ELMOD2	0,289071845	0,03079044	ELMO domain-containing protein 2 [Uniprot Acc. Q08DZ3];
ERAP1	0,555644922	0,001121149	Aminopeptidase [Uniprot Acc. A7MB81];
ERCC3	0,18506148	0,02651363	TFIIH basal transcription factor complex helicase XPB subunit [Uniprot Acc. Q1RMT1];
ERLIN1	0,279835276	0,032379816	Uncharacterized protein [Uniprot Acc. G3N0U8];
ESF1	0,364324875	0,044149312	Uncharacterized protein [Uniprot Acc. E1BKI4];
FAM73A	0,361758531	0,030279575	
FAM91A1	0,211342166	0,048389636	
FEM1B	0,21767092	0,017799541	Uncharacterized protein [Uniprot Acc. F1N162];
FMO4	0,280376258	0,039649942	Dimethylaniline monooxygenase (N-oxide-forming) [Uniprot Acc. G5E5J8];
FMR1	0,237491204	0,027071215	Uncharacterized protein [Uniprot Acc. F1MXQ7];
FNDC3A	0,251892634	0,044872696	Uncharacterized protein [Uniprot Acc. E1B8X1];
FUBP3	0,22350296	0,03696259	FUBP3 protein [Uniprot Acc. A6QP83];
G2E3	0,298256954	0,043047712	Uncharacterized protein [Uniprot Acc. F1N5U1];
GEN1	0,404622713	0,005598871	
GIMAP2	0,477045543	0,008416072	
GIMAP8	0,282711988	0,040929206	
GOPC	0,220867748	0,014665989	
GPATCH2	0,237635712	0,029599818	
GPATCH2L	0,250439807	0,033516497	
GTF3C3	0,320771696	0,00324954	

GTPBP4	0,268300178	0,022224422	Nucleolar GTP-binding protein 1 [Uniprot Acc. F1MYW4];
GUCY1B3	0,387325627	0,041772534	Guanylate cyclase soluble subunit beta-1 [Uniprot Acc. P16068];
HACL1	0,23369341	0,046760001	HACL1 protein [Uniprot Acc. A5PJL6];
HBS1L	0,267076078	0,006242876	HBS1-like protein [Uniprot Acc. Q2KHZ2];
HELB	0,339316003	0,03988872	Uncharacterized protein [Uniprot Acc. G3MYV2];
HERC4	0,212019455	0,019087232	Uncharacterized protein [Uniprot Acc. E1BAV2]; Hect domain and RLD 4 [Uniprot Acc. Q08DB6];
HMGXB4	0,225338861	0,033725871	HMGXB4 protein [Uniprot Acc. A6QQT0]; Uncharacterized protein [Uniprot Acc. F1MNG0];
ICE2	0,41993764	0,00449913	Little elongation complex subunit 2 [Uniprot Acc. Q0VCQ7];
IMMP1L	0,318089762	0,043873118	Mitochondrial inner membrane protease subunit 1 [Uniprot Acc. Q0VCH2];
IMPG2	0,371226331	0,046927451	
INPP5F	0,259566858	0,021568296	
JADE1	0,242149957	0,043047712	Protein Jade-1 [Uniprot Acc. Q5E9T7];
KIAA1429	0,170809682	0,040474893	
KIAA1551	0,358461685	0,010015818	
KIAA1715	0,276509521	0,026168373	KIAA1715 [Uniprot Acc. Q0VD11];
KIF3A	0,30625692	0,026679446	
KLHDC10	0,191841318	0,025292595	Kelch domain-containing protein 10 [Uniprot Acc. Q0IIC2];
KLHL20	0,290427139	0,003156107	Kelch-like protein 20 [Uniprot Acc. Q08DK3];
KLHL24	0,277352566	0,004735247	
KLHL9	0,252949252	0,02781027	Kelch-like protein 9 [Uniprot Acc. Q2T9Z7];
LEMD3	0,222672282	0,030652155	
LMBRD2	0,420120256	0,018303645	
LOC100335357	0,387819816	0,001187	
LOC100847770	0,374028572	0,022224422	
LOC104968476	0,322934259	0,022224422	Uncharacterized protein [Uniprot Acc. E1BEJ5];
LOC104969719	0,257907756	0,027071215	
LOC107133035	0,358565089	0,029332873	
LOC107133179	0,438246925	0,003156107	
LOC511229	0,265509319	0,020853523	
LOC512464	0,297013903	0,027393361	Solute carrier family 11 (Proton-coupled divalent metal ion transporters), member 2
LOC512684	0,497631129	0,004940883	ZNF547 protein [Uniprot Acc. A7YY23];
LOC530973	0,323192452	0,026168373	Uncharacterized protein [Uniprot Acc. E1BKR7];
LOC784007	0,381085849	0,00901427	

LOC787858	0,463882801	0,015265662	
LOC789996	0,360554632	0,041772534	
LRCH3	0,240551346	0,023926585	
LRRCC1	0,389460643	0,003067332	
LSG1	0,203649491	0,015265662	Large subunit GTPase 1 homolog [Uniprot Acc. Q2YDM7];
LSM11	0,24998678	0,041671148	
LTV1	0,255754909	0,03079044	Protein LTV1 homolog [Uniprot Acc. Q0VC06];
MAP3K7	0,178517076	0,032108	Mitogen-activated protein kinase kinase kinase 7 [Uniprot Acc. A2VDU3];
METTL16	0,259975804	0,031083643	Methyltransferase-like protein [Uniprot Acc. E1BCU7];
MFN1	0,296786552	0,022224422	
MFSD8	0,264380228	0,013781725	Uncharacterized protein [Uniprot Acc. E1BPY5];
MPP5	0,225067094	0,021101181	Uncharacterized protein [Uniprot Acc. E1BIQ8];
MRS2	0,270910442	0,024549387	MRS2 protein [Uniprot Acc. A6QR45];
MSANTD2	0,308685082	0,025511872	
MTMR2	0,292402577	0,023926585	Myotubularin-related protein 2 [Uniprot Acc. A6QLT2];
MYEF2	0,337163302	0,039791791	MYEF2 protein [Uniprot Acc. A6QQP0];
MYNN	0,252456356	0,013954817	Myoneurin [Uniprot Acc. Q3B7N9];
NAE1	0,286156122	0,031587073	NEDD8-activating enzyme E1 regulatory subunit [Uniprot Acc. E1B8X4];
NDC1	0,280792575	0,039791791	
NDUFAF7	0,219931738	0,040530844	Protein arginine methyltransferase NDUFAF7, mitochondrial [Uniprot Acc. Q2KHV5];
NEK4	0,340048011	0,001658985	NEK4 protein [Uniprot Acc. A6QQ15];
NEMF	0,242031013	0,01637913	
NKTR	0,232436381	0,039353831	Uncharacterized protein [Uniprot Acc. E1BJT5];
NOC3L	0,265189958	0,046458864	
NOL8	0,294317725	0,032108	Uncharacterized protein [Uniprot Acc. F1MRB9];
NPAT	0,25216102	0,04430762	
NSUN6	0,385633406	0,012052631	NSUN6 protein [Uniprot Acc. A4IF64];
OCIAD1	0,192200954	0,03894206	OCIA domain-containing protein 1 [Uniprot Acc. Q5E948]; Uncharacterized protein
PAXBP1	0,286669819	0,017036258	Uncharacterized protein [Uniprot Acc. F1MBJ4]; GC-rich sequence DNA-binding factor homolog
PCCA	0,206962253	0,048959753	PCCA protein [Uniprot Acc. A4FV90];
PCM1	0,251358023	0,012038354	
PDK1	0,424363909	0,013954817	Uncharacterized protein [Uniprot Acc. E1B8C5];
PDS5B	0,203883929	0,039293874	

PHTF2	0,353975649	0,002350951	Uncharacterized protein [Uniprot Acc. F1MNW1];
PIGA	0,342085114	0,031587073	Uncharacterized protein [Uniprot Acc. E1B7B9];
PIK3C3	0,246064134	0,022224422	Phosphatidylinositol 3-kinase [Uniprot Acc. A5PJQ9];
PKN2	0,328454726	0,006633314	Uncharacterized protein [Uniprot Acc. F1MFK1];
PPIP5K2	0,261425304	0,010802279	
PRPF39	0,344994647	0,017178777	PRPF39 protein [Uniprot Acc. A8E4M9];
PSIP1	0,200095143	0,023926585	PC4 and SFRS1-interacting protein [Uniprot Acc. Q8MJG1];
PTAR1	0,327160039	0,018377965	
RASA1	0,233361656	0,022224422	Ras GTPase-activating protein 1 [Uniprot Acc. P09851];
RBAK	0,357209145	0,005698498	Uncharacterized protein [Uniprot Acc. F1MHZ4];
RBM26	0,255414636	0,025322693	
RBM39	0,192515694	0,038179021	RBM39 protein [Uniprot Acc. Q2HJD8];
RBM4B	0,249010216	0,03894206	RNA binding motif protein 4B [Uniprot Acc. Q2KIV3];
RECQL	0,312283848	0,006295835	RecQ protein-like (DNA helicase Q1-like) [Uniprot Acc. A0JN36];
REV3L	0,210282343	0,030822757	
RFT1	0,29593755	0,010802279	Uncharacterized protein [Uniprot Acc. E1BNC6];
RFX3	0,26976168	0,045474325	
RIC8B	0,21411612	0,046458864	Uncharacterized protein [Uniprot Acc. E1BD35];
RNF168	0,251154907	0,026346998	E3 ubiquitin-protein ligase RNF168 [Uniprot Acc. F1MSN8];
RPP14	0,195235941	0,031156079	Ribonuclease P/MRP 14kDa subunit [Uniprot Acc. A0JN93];
RPS6KA3	0,139790334	0,02781027	Ribosomal protein S6 kinase [Uniprot Acc. A5PJL4];
RRN3	0,240102572	0,04251906	
RUFY2	0,510346385	0,000136201	
SACM1L	0,171511476	0,04430762	Phosphatidylinositide phosphatase SAC1 [Uniprot Acc. A6QL88];
SAMD8	0,288495519	0,013394046	
SASS6	0,276313071	0,046458864	Uncharacterized protein [Uniprot Acc. E1BAS3];
SBNO1	0,212755263	0,031041868	
SC5D	0,262938692	0,001658985	Sterol-C5-desaturase (ERG3 delta-5-desaturase homolog, S. cerevisiae)-like [Uniprot Acc. Q3SYX8];
SCAI	0,376061246	0,023948508	
SENP5	0,242074234	0,008862316	Uncharacterized protein [Uniprot Acc. F1MBS6];
SENP6	0,227807298	0,016916162	
SENP7	0,2224441	0,043047712	Sentrin-specific protease 7 [Uniprot Acc. A7MBJ2];
SEPSECS	0,42783235	0,002555525	O-phosphoseryl-tRNA(Sec) selenium transferase [Uniprot Acc. E1BPY3];

SERINC1	0,261107305	0,006183858	Serine incorporator 1 [Uniprot Acc. Q3MHV9];
SETX	0,244801973	0,004916491	
SFXN1	0,260060119	0,028526384	Sideroflexin [Uniprot Acc. A0A1P8NW48]; Sideroflexin-1 [Uniprot Acc. Q5E9M8];
SHPRH	0,303963284	0,047214469	
SIRT1	0,210781261	0,026346998	
SKIV2L2	0,265924145	0,017428746	SKIV2L2 protein [Uniprot Acc. A5PJZ8];
SLC25A36	0,293047636	0,022224422	Uncharacterized protein [Uniprot Acc. F1MU04];
SLC25A46	0,197333363	0,014349734	
SLC26A2	0,381650489	0,003465745	Sulfate transporter [Uniprot Acc. Q9BEG8];
SLC30A6	0,245311198	0,04430762	Zinc transporter 6 [Uniprot Acc. Q0VC54];
SLC30A9	0,259750628	0,022224422	Uncharacterized protein [Uniprot Acc. F1MDV2];
SLC35A3	0,262927047	0,031156079	UDP-N-acetylglucosamine transporter [Uniprot Acc. Q6YC49];
SLU7	0,271277232	0,041863416	Pre-mRNA-splicing factor SLU7 [Uniprot Acc. Q3ZBE5];
SMC3	0,265337214	0,026366809	Structural maintenance of chromosomes protein 3 [Uniprot Acc. O97594];
SMC4	0,236349867	0,04650335	Structural maintenance of chromosomes protein [Uniprot Acc. E1BMZ9];
SMURF2	0,193629569	0,026346998	
SNAPC3	0,331753792	0,022224422	Small nuclear RNA activating complex, polypeptide 3, 50kDa [Uniprot Acc. Q2TBW3];
SNRNP48	0,257690173	0,026346998	Uncharacterized protein [Uniprot Acc. E1B7L0];
SOCS4	0,279231767	0,048988811	Suppressor of cytokine signaling 4 [Uniprot Acc. Q0VC91];
SORBS1	0,431585766	0,010910871	Sorbin and SH3 domain containing 1 [Uniprot Acc. Q0VCF1];
SPICE1	0,369985808	0,000136201	Spindle and centriole-associated protein 1 [Uniprot Acc. Q2T9X8];
SPRTN	0,172850174	0,045358073	SprT-like domain-containing protein Spartan [Uniprot Acc. A5D979];
SREK1	0,288499096	0,025960223	
SREK1IP1	0,275709751	0,032656822	SFRS12IP1 protein [Uniprot Acc. Q17Q99];
SRSF11	0,269192088	0,046458864	Splicing factor, arginine/serine-rich 11 [Uniprot Acc. Q148M1];
ST3GAL6	0,286430446	0,004916491	Type 2 lactosamine alpha-2,3-sialyltransferase [Uniprot Acc. Q6H8M7];
SUCO	0,239156612	0,028713552	
TARDBP	0,206335001	0,03079044	Uncharacterized protein [Uniprot Acc. G3MX91];
TBCEL	0,282298617	0,048816049	
THUMPD1	0,281514729	0,03079044	THUMP domain-containing protein 1 [Uniprot Acc. Q24K03];
TIA1	0,241138587	0,038491543	TIA1 cytotoxic granule-associated RNA binding protein [Uniprot Acc. Q0VBZ6];
TLR3	0,343921609	0,03079044	Toll-like receptor 3 [Uniprot Acc. Q5TJ59];
TMED5	0,259403089	0,045358073	Transmembrane emp24 domain-containing protein 5 [Uniprot Acc. Q2KJ84];

TRAPPC8	0,194168699	0,040950983	
TRIM23	0,292464269	0,048979231	Uncharacterized protein [Uniprot Acc. F2Z4H6];
TRIP11	0,205442831	0,041359188	
TRMT13	0,384768551	0,002830845	Uncharacterized protein [Uniprot Acc. F6R1G6];
TTBK2	0,367267395	0,026346998	Uncharacterized protein [Uniprot Acc. E1BJC2];
TTC14	0,335006274	0,015616764	TTC14 protein [Uniprot Acc. A6QNT7];
TTC21B	0,404141162	0,0153092	
TTF1	0,201759123	0,026168373	TTF1 protein [Uniprot Acc. A6QNY0];
TUBGCP5	0,305925056	0,038179021	Gamma-tubulin complex component [Uniprot Acc. A6H7H5];
U2SURP	0,298469359	0,02487718	
UBA6	0,262748807	0,012052631	UBA6 protein [Uniprot Acc. A4FV03];
USP16	0,19557186	0,041273634	Ubiquitin carboxyl-terminal hydrolase 16 [Uniprot Acc. Q08DA3];
USP37	0,228101946	0,016685488	Ubiquitin carboxyl-terminal hydrolase 37 [Uniprot Acc. F1N5V1];
UTP20	0,299975301	0,048793192	
VAMP4	0,211653312	0,022582063	Vesicle-associated membrane protein 4 [Uniprot Acc. Q32L97];
VPS13A	0,348688729	0,01541584	
VPS50	0,293020868	0,030290693	
WDR11	0,18820734	0,039001691	BRWD2 protein [Uniprot Acc. A6QLS8];
YTHDC1	0,23501755	0,009560334	YTH domain containing 1 [Uniprot Acc. Q29RQ7]; Uncharacterized protein [Uniprot Acc. F1MIW9];
YTHDC2	0,252250301	0,034608365	Uncharacterized protein [Uniprot Acc. F1MNU7];
ZAR1L	0,304643026	0,038179021	
ZBTB26	0,32614915	0,037189721	
ZBTB6	0,450541886	0,006242876	Zinc finger and BTB domain-containing protein 6 [Uniprot Acc. Q0V8G8];
ZCCHC8	0,201034402	0,022224422	
ZDHHC17	0,327901242	0,019087232	
ZFP90	0,310935599	0,047600223	Uncharacterized protein [Uniprot Acc. E1B960];
ZFX	0,204496596	0,010075895	Zinc finger X-chromosomal protein [Uniprot Acc. O62836];
ZKSCAN8	0,292713844	0,026346998	ZNF192 protein [Uniprot Acc. A6QQG2];
ZMAT3	0,296538863	0,021402138	Zinc finger matrin-type protein 3 [Uniprot Acc. Q0IIC4];
ZNF12	0,35536094	0,006183858	Zinc finger protein 12 [Uniprot Acc. A8KC86];
ZNF131	0,266651572	0,004916491	ZNF131 protein [Uniprot Acc. A6QR64];
ZNF148	0,21063667	0,023299403	Zinc finger protein 148 [Uniprot Acc. Q3Y4E1];
ZNF154	0,338313942	0,039353831	

ZNF175	0,441673119	0,005308651	
ZNF182	0,242413082	0,040530844	Zinc finger protein 182 [Uniprot Acc. Q29RN3];
ZNF184	0,28598554	0,03820455	Zinc finger protein 184 [Uniprot Acc. A6QLU5];
ZNF248	0,267836831	0,02618868	Uncharacterized protein [Uniprot Acc. F1MTF3];
ZNF280D	0,26853098	0,022224422	
ZNF286A	0,401589026	0,004916491	ZNF286A protein [Uniprot Acc. A8WFL9]; Uncharacterized protein [Uniprot Acc. F1MTK8];
ZNF287	0,283360512	0,029332873	
ZNF436	0,275497807	0,028526384	Uncharacterized protein [Uniprot Acc. E1BMZ4];
ZNF451	0,215639603	0,048199854	Uncharacterized protein [Uniprot Acc. E1BGM1];
ZNF518A	0,446198294	4,16985E-06	Uncharacterized protein [Uniprot Acc. E1BG18];
ZNF572	0,298015927	0,014665989	Zinc finger protein 572 [Uniprot Acc. Q32KN0];
ZNF605	0,421089325	0,003579557	
ZNF624	0,352321721	0,030894473	
ZNF644	0,192562014	0,049468153	ZNF644 protein [Uniprot Acc. A3KMW9];
ZNF655	0,214823769	0,020937756	Zinc finger protein 655 [Uniprot Acc. Q0IIK3];
ZNF830	0,233295449	0,026739364	ZNF830 protein [Uniprot Acc. A3KN01];
ZNF93	0,277486117	0,046645177	Uncharacterized protein [Uniprot Acc. F1MEQ1];
ZRANB2	0,251312836	0,026366809	Zinc finger Ran-binding domain-containing protein 2 [Uniprot Acc. A7YWH2];
ZZZ3	0,343205162	0,001658985	ZZZ3 protein [Uniprot Acc. A6QLC9];
AARS	-0,17049075	0,015277535	AARS protein [Uniprot Acc. A6QLT9];
ABCD1	-0,32213391	0,013394046	ATP-binding cassette, sub-family D (ALD), member 1 [Uniprot Acc. Q2KJ57];
ACTB	-0,24493631	0,00090522	Actin, cytoplasmic 1 [Uniprot Acc. P60712];
ACTG1	-0,2338409	0,046458864	Actin, cytoplasmic 2 [Uniprot Acc. P63258];
ADRM1	-0,19421825	0,013954817	Proteasomal ubiquitin receptor ADRM1 [Uniprot Acc. A1L5A6];
AKT1	-0,24122065	0,020488753	RAC-alpha serine/threonine-protein kinase [Uniprot Acc. Q01314];
AKT2	-0,23417617	0,014927954	Uncharacterized protein [Uniprot Acc. E1B9D1];
ALDH16A1	-0,2081761	0,046458864	Aldehyde dehydrogenase family 16 member A1 [Uniprot Acc. A6QR56];
ANAPC11	-0,29127577	0,04704223	Anaphase-promoting complex subunit 11 [Uniprot Acc. Q3ZCF6];
AP2A1	-0,31727151	0,033590764	Adaptor-related protein complex 2, short form [Uniprot Acc. Q1JPJ7];
APRT	-0,31561993	0,003939688	Adenine phosphoribosyltransferase [Uniprot Acc. Q56JW4];
ARF6	-0,27685816	0,034980826	Uncharacterized protein [Uniprot Acc. G3N3N1];
ARHGAP1	-0,30446433	0,027393361	Uncharacterized protein [Uniprot Acc. F6RWK1];
ARHGDIA	-0,21806359	0,014755658	Rho GDP-dissociation inhibitor 1 [Uniprot Acc. P19803];

ARPC1B	-0,23918443	0,044437603	Actin-related protein 2/3 complex subunit 1B [Uniprot Acc. Q58CQ2];
ARRDC1	-0,28595901	0,034854118	Arrestin domain containing 1 [Uniprot Acc. Q0P5B0];
ARSA	-0,31378274	0,00090522	Arylsulfatase A [Uniprot Acc. Q08DD1];
ASS1	-0,35356678	0,047214469	Argininosuccinate synthase [Uniprot Acc. P14568];
ATG2A	-0,33587146	0,041359188	
ATG4D	-0,3898867	0,000173468	Cysteine protease [Uniprot Acc. A5PK87];
ATP5D	-0,36944141	0,0153092	ATP synthase subunit delta, mitochondrial [Uniprot Acc. P05630];
ATP5G2	-0,1877398	0,030840927	ATP synthase F(0) complex subunit C2, mitochondrial [Uniprot Acc. P07926];
ATP6V1F	-0,31895118	0,048199854	V-type proton ATPase subunit F [Uniprot Acc. Q28029];
ATXN7L3	-0,16573514	0,026346998	Ataxin-7-like protein 3 [Uniprot Acc. E1BAT0];
BAG6	-0,16750778	0,030458606	Uncharacterized protein [Uniprot Acc. F1MY28];
BAK1	-0,31361321	0,005598871	BCL2-antagonist/killer 1 [Uniprot Acc. Q05KI7];
BANF1	-0,1998516	0,023206764	Barrier-to-autointegration factor [Uniprot Acc. P61283];
BCKDHA	-0,23185979	0,048188741	2-oxoisovalerate dehydrogenase subunit alpha, mitochondrial [Uniprot Acc. P11178];
BCL9L	-0,27739934	0,040530844	Uncharacterized protein [Uniprot Acc. E1B8K0];
BCORL1	-0,28480915	0,031435173	Uncharacterized protein [Uniprot Acc. F1MML3];
BLVRB	-0,32449315	0,046342544	Flavin reductase (NADPH) [Uniprot Acc. P52556];
BoLA	-0,34594502	0,017890047	BoLA protein [Uniprot Acc. A7YWH4];
BOLA-DRB3	-0,32593107	0,036668985	
BORCS6	-0,35814957	0,01501894	BLOC-1-related complex subunit 6 [Uniprot Acc. Q3SX20];
C11H9orf142	-0,31328993	0,048188741	C11H9ORF142 protein [Uniprot Acc. A6QQL6];
CAMK2N1	-0,4245637	0,046945865	Calcium/calmodulin-dependent protein kinase II inhibitor 1 [Uniprot Acc. A7MBG3];
CAPNS1	-0,23007516	0,010975502	Calpain small subunit 1 [Uniprot Acc. P13135];
CARM1	-0,21223892	0,013781725	
CCDC97	-0,29108439	0,006183858	CCDC97 protein [Uniprot Acc. A3KMW0];
CD37	-0,27336679	0,010802279	Leukocyte antigen CD37 [Uniprot Acc. Q2KHY8];
CD81	-0,23021208	0,010602731	CD81 antigen [Uniprot Acc. Q3ZCD0];
CDK9	-0,18667211	0,015277535	Cyclin-dependent kinase 9 [Uniprot Acc. Q5EAB2];
CFL1	-0,1906739	0,013954817	Cofilin 1 (Non-muscle) [Uniprot Acc. B0JYL8]; Cofilin-1 [Uniprot Acc. Q5E9F7];
CHERP	-0,27316856	0,01659963	CHERP protein [Uniprot Acc. A8E648];
CHST12	-0,30201376	0,049856002	Carbohydrate sulfotransferase [Uniprot Acc. Q08DS2];
CIC	-0,31483929	0,026346998	Uncharacterized protein [Uniprot Acc. G3MX46];
CLPTM1	-0,20502743	0,006183858	Cleft lip and palate transmembrane protein 1 homolog [Uniprot Acc. Q2NL17];

COPG1	-0,19272108	0,029332873	Coatomer subunit gamma [Uniprot Acc. A0A140T886]; Coatomer subunit gamma-1
CORO1A	-0,22322909	0,01659963	Coronin-1A [Uniprot Acc. Q92176];
COX8A	-0,24804429	0,015265662	Cytochrome c oxidase subunit 8A, mitochondrial [Uniprot Acc. P14622];
CRKL	-0,30417268	0,031587073	
CRTC2	-0,28521526	0,00324954	CREB-regulated transcription coactivator 2 [Uniprot Acc. Q08E26];
CSK	-0,21819538	0,000987124	Tyrosine-protein kinase CSK [Uniprot Acc. Q0VBZ0];
CSNK2B	-0,22689071	0,001008407	Casein kinase II subunit beta [Uniprot Acc. N0E640];
CSRP1	-0,25055716	0,037116475	Cysteine and glycine-rich protein 1 [Uniprot Acc. Q3MHY1];
CTDSP1	-0,27670786	0,024171389	Uncharacterized protein [Uniprot Acc. E1BDE3];
CYSTM1	-0,37078502	0,022224422	Cysteine-rich and transmembrane domain-containing protein 1 [Uniprot Acc. Q32LK2];
DEDD	-0,16782826	0,021101181	Death effector domain containing [Uniprot Acc. Q3ZCB7]; Uncharacterized protein
DGKZ	-0,22000654	0,045358073	Putative uncharacterized protein [Uniprot Acc. A7E3C6];
DOK1	-0,16194874	0,041292744	Docking protein 1 [Uniprot Acc. Q5EA84];
DPF2	-0,14436112	0,039791791	D4, zinc and double PHD fingers family 2 [Uniprot Acc. A6QQS0];
DPP3	-0,21082279	0,040474893	Dipeptidyl peptidase 3 [Uniprot Acc. Q58CS3];
DPP9	-0,20562153	0,041462937	
EFHD2	-0,32148568	0,032108	EF-hand domain-containing protein D2 [Uniprot Acc. A5D7A0];
EIF3L	-0,1113013	0,048723792	Eukaryotic translation initiation factor 3 subunit L [Uniprot Acc. Q3ZCK1];
EIF5A	-0,22030892	0,010075895	Eukaryotic translation initiation factor 5A-1 [Uniprot Acc. Q6EWQ7];
EMD	-0,25355773	0,000136201	Emerin [Uniprot Acc. Q6XZP8];
ENDOG	-0,30906778	0,048021019	Endonuclease G, mitochondrial [Uniprot Acc. P38447];
ENO1	-0,26221198	0,007023524	Alpha-enolase [Uniprot Acc. Q9XSJ4];
EPN1	-0,27890352	0,013954817	Uncharacterized protein [Uniprot Acc. F1N579];
ERCC2	-0,26009282	0,023926585	TFIIH basal transcription factor complex helicase XPD subunit [Uniprot Acc. A6QLJ0];
ERF	-0,36452878	0,003015023	Uncharacterized protein [Uniprot Acc. F1MET2];
FAM53B	-0,27030442	0,022224422	
FBRS	-0,30386004	0,032348363	
FKBP8	-0,21885276	0,023926585	Peptidylprolyl isomerase [Uniprot Acc. F1N2P6];
FLII	-0,22165062	0,048988811	
FLYWCH2	-0,29957439	0,018022823	FLYWCH family member 2-like [Uniprot Acc. E1BBK3];
FTH1	-0,468414	0,003480158	Ferritin heavy chain [Uniprot Acc. O46414];
GATAD2A	-0,18821801	0,016065342	GATAD2A protein [Uniprot Acc. A7YY50];
GFER	-0,3481381	0,024072064	Sulfhydryl oxidase [Uniprot Acc. E1BF52];

GLRX	-0,28568578	0,021101181	Glutaredoxin-1 [Uniprot Acc. P10575];
GNB2	-0,31039916	0,004916491	Guanine nucleotide-binding protein G(I)/G(S)/G(T) subunit beta-2 [Uniprot Acc. P11017];
GPI	-0,20690662	0,010802279	Glucose-6-phosphate isomerase [Uniprot Acc. Q3ZBD7];
GPX1	-0,31421541	0,039353831	Glutathione peroxidase 1 [Uniprot Acc. P00435];
GSK3A	-0,25630891	0,026421435	GSK3A protein [Uniprot Acc. A6QLB8];
GSN	-0,38971268	0,029344097	Gelsolin [Uniprot Acc. Q3SX14];
GYS1	-0,37451891	0,046458864	Glycogen (starch) synthase, muscle [Uniprot Acc. A7MB78];
H2AFJ	-0,26132406	0,045358073	Histone H2A.J [Uniprot Acc. Q3ZBX9];
HDLBP	-0,19962741	0,039642083	
HEXIM1	-0,25693547	0,013630063	Protein HEXIM1 [Uniprot Acc. Q0X0C4];
HK1	-0,266649	0,030822757	Hexokinase 1 [Uniprot Acc. Q5W5U3];
HMG20B	-0,23437733	0,046458864	SWI/SNF-related matrix-associated actin-dependent regulator of chromatin subfamily E member 1
HMGA1	-0,30042342	0,006242876	High mobility group AT-hook 1 [Uniprot Acc. Q0VC27];
HPCAL1	-0,30956086	0,016685488	Hippocalcin-like protein 1 [Uniprot Acc. P29105];
HSF1	-0,19051807	0,029023986	Heat shock factor protein 1 [Uniprot Acc. Q08DJ8];
HSPB1	-0,32399776	0,044437603	Heat shock protein beta-1 [Uniprot Acc. Q3T149];
IDH2	-0,20284888	0,011234187	Isocitrate dehydrogenase (NADP), mitochondrial [Uniprot Acc. Q04467];
IFI30	-0,34287318	0,044437603	Gamma-interferon-inducible lysosomal thiol reductase [Uniprot Acc. A6QPN6];
IFI6	-0,49709146	0,013781725	Interferon alpha-inducible protein 6 [Uniprot Acc. Q6IED8];
IL17RA	-0,29527674	0,029023986	
IL2RG	-0,19697098	0,028526384	Cytokine receptor common subunit gamma [Uniprot Acc. Q95118];
INTS5	-0,28650369	0,021248955	Integrator complex subunit 5 [Uniprot Acc. A1L552];
IRF2BP1	-0,29646252	0,022224422	
ITM2C	-0,27994835	0,026168373	Integral membrane protein 2C [Uniprot Acc. A2VDN0];
KCNAB2	-0,27455836	0,031156079	Voltage-gated potassium channel subunit beta-2 [Uniprot Acc. Q27955];
KCTD11	-0,3118033	0,033028365	BTB/POZ domain-containing protein KCTD11 [Uniprot Acc. F2Z4C0];
KEAP1	-0,25870842	0,004087483	KEAP1 protein [Uniprot Acc. A7MBG4];
KLF16	-0,288965	0,029332873	
LAMTOR2	-0,3396021	0,03820455	Ragulator complex protein LAMTOR2 [Uniprot Acc. Q3T132];
LIMK1	-0,284552	0,022224422	Uncharacterized protein [Uniprot Acc. E1BC64];
LOC100126544	-0,35230139	0,013233085	LOC100126544 protein [Uniprot Acc. A8QIC0];
LOC100336747	-0,34525369	0,043047712	
LOC104974875	-0,37536433	0,048199854	

LOC107131224	-0,46887621	0,029510011	
LOC107132738	-0,28670599	0,018377965	
LOC530437	-0,27033349	0,031587073	
LOC540981	-0,28732993	0,041292744	
LOC616200	-0,48268065	0,001806346	
LPCAT3	-0,32143357	0,015701994	Lysophospholipid acyltransferase 5 [Uniprot Acc. Q3SZL3];
LRRC41	-0,18654996	0,049856002	Leucine-rich repeat-containing protein 41 [Uniprot Acc. Q29RR1];
LSP1	-0,25167298	4,09829E-05	Lymphocyte-specific protein 1 [Uniprot Acc. Q0P5E0];
LYL1	-0,30524042	0,000136201	Uncharacterized protein [Uniprot Acc. E1BAR3];
LYPLA2	-0,26158542	0,025292595	Lysophospholipase II [Uniprot Acc. Q17QL8];
MAF1	-0,20104924	0,017428746	Repressor of RNA polymerase III transcription MAF1 [Uniprot Acc. Q3T0W8];
MAFK	-0,2641307	0,020672009	Uncharacterized protein [Uniprot Acc. E1BB01];
MAP2K3	-0,21929174	0,026346998	MAP2K3 protein [Uniprot Acc. A4IFH7];
MAP3K11	-0,33108776	0,04637804	MAP3K11 protein [Uniprot Acc. A6QQU8];
MAP7D1	-0,33516338	0,008862316	
MAZ	-0,21993132	0,032348363	
MED16	-0,24939438	0,009550214	
MED25	-0,22240739	0,032108	Mediator of RNA polymerase II transcription subunit 25 [Uniprot Acc. A2VE44];
MFSD5	-0,26872824	0,001695497	Molybdate-anion transporter [Uniprot Acc. Q0VC03];
MID1IP1	-0,25760861	0,026346998	MID1 interacting protein 1 (Gastrulation specific G12 homolog (Zebrafish)) [Uniprot Acc. Q08E46];
MIF	-0,39954978	0,026346998	Macrophage migration inhibitory factor [Uniprot Acc. A0A0F7RPX0];
MLF2	-0,22465296	0,036091784	
MOB3A	-0,22112132	0,018814082	MOB kinase activator 3A [Uniprot Acc. Q58D63];
MOSPD3	-0,24493237	0,042674352	Motile sperm domain-containing protein 3 [Uniprot Acc. Q3T033];
MRPL12	-0,34722464	0,006866404	39S ribosomal protein L12, mitochondrial [Uniprot Acc. Q7YR75];
MRPL36	-0,30078775	0,013954817	Ribosomal protein [Uniprot Acc. G3MYN6];
MRPS34	-0,28712066	0,039353831	28S ribosomal protein S34, mitochondrial [Uniprot Acc. P82929];
MTX1	-0,24292879	0,040530844	Metaxin-1 [Uniprot Acc. Q2TBS1];
MXD3	-0,3776068	0,042211969	Uncharacterized protein [Uniprot Acc. E1BBZ1];
NACC1	-0,24434022	0,002204505	Uncharacterized protein [Uniprot Acc. E1BQ03];
NCOR2	-0,30411119	0,03696259	
NDUFA7	-0,25640248	0,01781997	NADH dehydrogenase (ubiquinone) 1 alpha subcomplex subunit 7 [Uniprot Acc. Q05752];
NDUFB7	-0,31478525	0,014122844	NADH dehydrogenase (ubiquinone) 1 beta subcomplex subunit 7 [Uniprot Acc. Q02368];

NDUFS6	-0,21640404	0,04430762	NADH dehydrogenase (ubiquinone) iron-sulfur protein 6, mitochondrial [Uniprot Acc. P23934];
NDUFS7	-0,22600294	0,03079044	NADH dehydrogenase (ubiquinone) iron-sulfur protein 7, mitochondrial [Uniprot Acc. P42026];
NDUFS8	-0,30030407	0,023926585	NADH dehydrogenase (ubiquinone) iron-sulfur protein 8, mitochondrial [Uniprot Acc. P42028];
NECAP2	-0,20921475	0,025322693	Adaptin ear-binding coat-associated protein 2 [Uniprot Acc. Q5E9Q4];
NFKBIB	-0,36503669	0,000422931	
NR1H2	-0,15489048	0,022224422	Nuclear receptor subfamily 1, group H, member 2 [Uniprot Acc. Q58CP4];
NRM	-0,36077715	0,008989393	Nurim [Uniprot Acc. Q32LM8];
NUP62	-0,25626406	0,001187	
OCEL1	-0,33230893	0,013233085	Uncharacterized protein [Uniprot Acc. F6RA30];
OGFR	-0,22213512	0,006183858	Opioid growth factor receptor [Uniprot Acc. Q2HJD3];
ORAI1	-0,30448101	0,006433501	ORAI1 protein [Uniprot Acc. A5PKA7];
OTUB1	-0,17101299	0,026346998	Ubiquitin thioesterase [Uniprot Acc. Q3T0Y1];
PCBP1	-0,1764423	0,008862316	Poly(rC)-binding protein 1 [Uniprot Acc. Q5E9A3];
PCIF1	-0,24853516	0,000987124	
PDXP	-0,36941732	0,018843832	Pyridoxal phosphate phosphatase [Uniprot Acc. Q3ZBF9];
PFKL	-0,20520825	0,031243093	ATP-dependent 6-phosphofructokinase, liver type [Uniprot Acc. A1A4J1];
PFN1	-0,36328627	0,006433501	Profilin-1 [Uniprot Acc. P02584];
PINK1	-0,1849626	0,032348363	PINK1 protein [Uniprot Acc. A5PJP5];
PLEKHO2	-0,27071543	0,01230714	
PNPLA2	-0,35517254	0,017799541	Adipose triglyceride lipase [Uniprot Acc. D6BQM3];
POLD1	-0,29373784	0,03820455	DNA polymerase delta catalytic subunit [Uniprot Acc. P28339];
POLR2A	-0,38325086	0,002555525	
PPDPF	-0,35115126	0,028159697	Pancreatic progenitor cell differentiation and proliferation factor [Uniprot Acc. Q3ZCB6];
PPM1F	-0,25362892	0,048988811	
PPP1R18	-0,19555625	0,017134133	KIAA1949 [Uniprot Acc. Q08DC3];
PPP1R9B	-0,19899006	0,022224422	
PPP2R1A	-0,24064656	0,018046883	Serine/threonine-protein phosphatase 2A 65 kDa regulatory subunit A alpha isoform
PPP2R4	-0,20613838	0,046342544	Serine/threonine-protein phosphatase 2A activator [Uniprot Acc. Q2KJ44];
PPP4C	-0,16981299	0,032108	Serine/threonine-protein phosphatase 4 catalytic subunit [Uniprot Acc. A6H772];
PPP6R1	-0,31493281	0,042383631	
PRAM1	-0,46932101	0,015265662	
PRR12	-0,24678463	0,049432741	
PRR3	-0,2403481	0,03079044	Proline rich 3 [Uniprot Acc. Q3MHG5];

PRSS2	-0,27328859	0,026346998	Pancreatic anionic trypsinogen [Uniprot Acc. Q547S4];
PSENEN	-0,25890747	0,023352331	Gamma-secretase subunit PEN-2 [Uniprot Acc. Q5G235];
PSMB10	-0,28212913	2,16987E-06	Proteasome subunit beta type-10 [Uniprot Acc. Q3T0T1];
PSMB4	-0,2947405	0,035441833	Proteasome subunit beta type-4 [Uniprot Acc. Q3T108];
PSMB8	-0,2070237	0,015616764	Proteasome subunit beta type-8 [Uniprot Acc. Q3T112];
PSMB9	-0,23410809	0,043047712	Proteasome subunit beta type-9 [Uniprot Acc. Q3SZC2];
PSMD4	-0,13495617	0,046458864	26S proteasome non-ATPase regulatory subunit 4 [Uniprot Acc. Q58DA0];
PTMS	-0,46370879	0,006242876	
PXN	-0,37987299	0,02987922	
RAB1B	-0,30452406	0,006242876	Ras-related protein Rab-1B [Uniprot Acc. Q2HJH2];
RAC2	-0,23869375	0,026168373	Ras-related C3 botulinum toxin substrate 2 (Rho family, small GTP binding protein Rac2)
RAD23A	-0,19382977	0,045358073	UV excision repair protein RAD23 homolog A [Uniprot Acc. A3KMV2];
RALY	-0,13993113	0,027175293	RNA binding protein (Autoantigenic, hnRNP-associated with lethal yellow) short isoform
RAPGEF1	-0,19391955	0,029344097	
RASSF5	-0,18009377	0,030458606	
RAVER1	-0,28717895	0,00189883	RAVER1 protein [Uniprot Acc. A4FUZ2];
RBCK1	-0,23605525	0,021101181	RanBP-type and C3HC4-type zinc finger containing 1 [Uniprot Acc. Q1JPC8];
RBM42	-0,23261779	0,001187	RNA-binding protein 42 [Uniprot Acc. Q0P5L0];
RCC2	-0,22258828	0,026168373	RCC2 protein [Uniprot Acc. A6QL85];
RFX2	-0,42308888	0,044437603	DNA-binding protein RFX2 [Uniprot Acc. A6QLW9];
RGS19	-0,29221648	0,017799541	Regulator of G-protein signaling 19 [Uniprot Acc. Q08DC7];
RHOG	-0,35231011	0,046458864	Ras homolog gene family, member G (Rho G) [Uniprot Acc. Q1RMI2];
RIN3	-0,2958865	0,032676101	
RPL28	-0,22828775	0,009158173	60S ribosomal protein L28 [Uniprot Acc. Q3T0L7];
RUNX3	-0,42977325	0,00143685	
SAP130	-0,2216188	0,031872948	
SASH3	-0,1775104	0,015889789	SAM and SH3 domain-containing protein 3 [Uniprot Acc. A0JN71];
SCAF1	-0,22508239	0,021170201	
SCAND1	-0,28444601	0,040950983	SCAN domain-containing protein 1 [Uniprot Acc. Q32PG5];
SCIN	-0,41252554	0,046458864	Adseverin [Uniprot Acc. Q28046];
SCLY	-0,27448426	0,031083643	Selenocysteine lyase [Uniprot Acc. A2VDS1];
SDHA	-0,10539803	0,039353831	Succinate dehydrogenase (ubiquinone) flavoprotein subunit, mitochondrial [Uniprot Acc. P31039];
SEPT9	-0,18896446	0,013992427	Uncharacterized protein [Uniprot Acc. F1N6U4];

SH3BGRL3	-0,33442225	0,004916491	SH3 domain-binding glutamic acid-rich-like protein 3 [Uniprot Acc. Q3ZCL8];
SH3BP1	-0,25176697	0,027268548	Uncharacterized protein [Uniprot Acc. E1BL56];
SHARPIN	-0,26712026	0,030822757	Sharpin [Uniprot Acc. E1BDF2];
SHKBP1	-0,18259357	0,015265662	SH3KBP1-binding protein 1 [Uniprot Acc. A3KMV1];
SIPA1	-0,33274587	0,04430762	SIPA1 protein [Uniprot Acc. A6QPH2];
SLC10A3	-0,25001273	0,016916162	P3 protein [Uniprot Acc. Q0V8N6];
SLC15A3	-0,36433813	0,021101181	Uncharacterized protein [Uniprot Acc. F1MYU7];
SLC25A1	-0,2781026	0,032656822	
SLC25A39	-0,19920398	0,049605369	Solute carrier family 25 member 39 [Uniprot Acc. Q17QI7];
SLC25A6	-0,31529665	0,004916491	ADP/ATP translocase 3 [Uniprot Acc. P32007];
SLC27A1	-0,25431106	0,002079474	Long-chain fatty acid transport protein 1 [Uniprot Acc. A4IFM2];
SLC9A3R1	-0,33565486	0,000173468	Na(+)/H(+) exchange regulatory cofactor NHE-RF1 [Uniprot Acc. Q3SZK8];
SMARCB1	-0,17564866	0,048988811	SWI/SNF-related matrix-associated actin-dependent regulator of chromatin
SOWAHD	-0,36895129	0,044423207	
SREBF2	-0,23497977	0,019770373	
SSU72	-0,17073535	0,046615775	RNA polymerase II subunit A C-terminal domain phosphatase SSU72 [Uniprot Acc. Q17QI2];
ST3GAL2	-0,22846531	0,043873118	ST3 beta-galactoside alpha-2,3-sialyltransferase 2 [Uniprot Acc. Q6H8M9];
STAT6	-0,18938371	0,044606792	Signal transducer and activator of transcription [Uniprot Acc. F1MGJ4];
SUPT4H1	-0,21486475	0,023843722	Transcription elongation factor SPT4 [Uniprot Acc. Q3SYX6];
SUV39H1	-0,24791807	0,048137454	Histone-lysine N-methyltransferase SUV39H1 [Uniprot Acc. Q2NL30];
SYN1	-0,36838097	0,046458864	Synapsin-1 [Uniprot Acc. P17599];
SYNGR2	-0,22960658	0,001187	Synaptogyrin-2 [Uniprot Acc. A7E3W5];
SZRD1	-0,18950415	0,046953609	
TAF10	-0,21220831	0,01230714	TAF10 protein [Uniprot Acc. A5PJW1];
TESK1	-0,31418812	0,001565614	Uncharacterized protein [Uniprot Acc. E1BPB5];
TGFB1	-0,28941058	0,028526384	Transforming growth factor beta-1 [Uniprot Acc. P18341];
TICAM1	-0,27794116	0,021170201	TIR domain-containing adapter molecule 1 [Uniprot Acc. Q4JF29];
TLN1	-0,27271293	0,046595736	
TMEM104	-0,27959775	0,026346998	Transmembrane protein 104 [Uniprot Acc. A0JNF7];
TMEM160	-0,3702172	0,042100333	Transmembrane protein 160 [Uniprot Acc. Q24JY6];
TNFAIP8L2	-0,19760582	0,047728386	Tumor necrosis factor alpha-induced protein 8-like protein 2 [Uniprot Acc. Q3ZBK5];
TNK2	-0,31780855	0,022141676	Activated CDC42 kinase 1 [Uniprot Acc. Q17R13];
TOB2	-0,23741955	0,001187	TOB2 protein [Uniprot Acc. A4FUY5];

TOR4A	-0,34920025	0,008793035	Uncharacterized protein [Uniprot Acc. G3X7J5];
TPI1	-0,23305329	0,031083643	Triosephosphate isomerase [Uniprot Acc. Q5E956];
TRIM8	-0,20973024	0,04840956	Uncharacterized protein [Uniprot Acc. E1BJC6];
TSC22D4	-0,29046774	0,009625321	TSC22 domain family, member 4 [Uniprot Acc. Q0IIB5];
TUFM	-0,16190398	0,039791791	Elongation factor Tu, mitochondrial [Uniprot Acc. P49410];
U2AF2	-0,21333036	0,040050604	U2 small nuclear RNA auxiliary factor 2 [Uniprot Acc. Q24JZ8];
UBALD1	-0,22225423	0,046458864	
UBE2M	-0,16603417	0,031156079	NEDD8-conjugating enzyme Ubc12 [Uniprot Acc. A3KN22];
UBE2O	-0,20837969	0,045358073	Uncharacterized protein [Uniprot Acc. F1N3I3];
UBE2S	-0,34044404	0,027069891	Ubiquitin-conjugating enzyme E2 S [Uniprot Acc. Q1RML1];
UCP2	-0,38983005	0,031243093	Mitochondrial uncoupling protein 2 [Uniprot Acc. Q3SZI5];
UQCC3	-0,37343399	0,026346998	Ubiquinol-cytochrome-c reductase complex assembly factor 3 [Uniprot Acc. Q148G8];
UQCR11	-0,25736742	0,031156079	Cytochrome b-c1 complex subunit 10 [Uniprot Acc. P07552];
USF2	-0,16261732	0,022224422	Upstream stimulatory factor 2 [Uniprot Acc. Q6XD34];
USP21	-0,21288291	0,046760001	Ubiquitin carboxyl-terminal hydrolase 21 [Uniprot Acc. Q2KJ72];
VASP	-0,31633395	0,046458864	Vasodilator-stimulated phosphoprotein [Uniprot Acc. Q2TA49];
VPS37C	-0,20336013	0,046458864	Uncharacterized protein [Uniprot Acc. E1BC70];
WDR1	-0,20724131	0,017434081	WD repeat-containing protein 1 [Uniprot Acc. Q2KJH4];
WDR81	-0,19918298	0,046878791	
YPEL3	-0,26540195	0,006002039	Protein yippee-like 3 [Uniprot Acc. A6QPH8];
ZBTB7A	-0,23014137	0,022224422	
ZBTB7B	-0,29899247	0,046760001	Uncharacterized protein [Uniprot Acc. E1BGW3];
ZGPAT	-0,21026254	0,048959753	Zinc finger CCCH-type with G patch domain-containing protein [Uniprot Acc. Q17QX2];
ZNF358	-0,35468208	0,022224422	Uncharacterized protein [Uniprot Acc. G5E6Q2];
ZNF385A	-0,3600844	0,031397128	
ZNF408	-0,27503901	0,016065342	Uncharacterized protein [Uniprot Acc. E1BCQ9];
ZNF574	-0,22655483	0,049468153	Zinc finger protein 574 [Uniprot Acc. Q29RK0];
ZNF687	-0,22825693	0,028700805	Uncharacterized protein [Uniprot Acc. E1BQ01];
ZNF865	-0,39448395	0,036798745	

Supplementary Table 4. Blood cell count comparison between control and I supplementation groups

Parameters	CTR		IG	
	**T0**	**T8**	**T0**	**T8**
WBC (*1000 μl^{-1})	8.19±1.29	7.98±0.25	8.25±0.23	8.14±0.47
Monocyte (%)	4.24±0.43	4.11±0.28	4.08±0.32	4.36±0.11
Lymphocyte (%)	50.98±3.69	52.25±4.40	51.77±2.24	52.85±2.25
Basophil (%)	1.10±0.18	1.03±0.10	1.17±0.05	1.12±0.20
Neutrophils (%)	38.22±2.99	37.52±2.51	36.28±3.79	38.86±2.37
Eosinophils (%)	4.39±0.52	4.51±0.47	4.36±0.59	4.16±0.32
